# The BEACON study: protocol for a cohort study as part of an evaluation of the effectiveness of smartphone-assisted problem-solving therapy in men who present with intentional self-harm to emergency departments in Ontario

**DOI:** 10.1186/s13063-020-04424-w

**Published:** 2020-11-13

**Authors:** Simon Hatcher, Marnin Heisel, Oyedeji Ayonrinde, Julie K. Campbell, Ian Colman, Daniel J. Corsi, Nicole E. Edgar, Lindsay Gillett, Sidney H. Kennedy, Sophia Lakatoo Hunt, Paul Links, Sarah MacLean, Viraj Mehta, Christopher Mushquash, Alicia Raimundo, Sakina J. Rizvi, Refik Saskin, Ayal Schaffer, Alaaddin Sidahmed, Mark Sinyor, Claudio Soares, Monica Taljaard, Valerie Testa, Kednapa Thavorn, Venkatesh Thiruganasambandamoorthy, Christian Vaillancourt

**Affiliations:** 1 Ottawa Hospital Research Institute, Ottawa, Canada; 2 University of Ottawa, Ottawa, Canada; 3 Lawson Health Research Institute, London, Canada; 4 Western University, London, Canada; 5 Queen’s University, Kingston, Canada; 6 Kingston Health Sciences Centre, Kingston, Canada; 7 Canadian Association for Suicide Prevention, Winnipeg, Canada; 8 Sioux Lookout Meno Ya Win Health Centre, Sioux Lookout, Canada; 9 St. Michael’s Hospital, Toronto, Canada; 10 University of Toronto, Toronto, Canada; 11 William Osler Health System, Toronto, Canada; 12 McMaster University, Hamilton, Canada; 13 Carleton University, Ottawa, Canada; 14 Lakehead University, Thunder Bay, Canada; 15 Institute for Clinical Evaluative Sciences, Toronto, Canada; 16 Sunnybrook Research Institute, Toronto, Canada; 17 Wayne State University, Detroit, USA

**Keywords:** Self-harm, Suicide, Problem-solving therapy, Blended care, Cognitive behaviour therapy, Men, Suicide prevention

## Abstract

**Background:**

Patients who present to emergency departments after intentional self-harm are at an increased risk of dying by suicide. This applies particularly to men, who represent nearly two-thirds of those who die by suicide in Ontario. One way of potentially addressing this gap is to offer a course of blended problem-solving therapy, comprised of a brief course of evidence-based psychotherapy for individuals at risk for suicide, facilitated by the use of a patient-facing smartphone application and a clinician-facing “dashboard.” This approach has the potential to combine the benefits of face-to-face therapy and technology to create a novel intervention.

**Methods:**

This is a cohort study nested within a larger pragmatic multicentre pre- and post-design cluster randomised trial. Suicidal ideation assessed by the Beck Scale for Suicide Ideation is the primary outcome variable. Secondary outcome measures include depression (Patient Health Questionnaire-9), anxiety (Generalized Anxiety Disorder 7-item scale), post-traumatic stress disorder (Primary Care PTSD Screen), health-related quality of life (EuroQol 5-dimension 5-level questionnaire), meaning in life (Experienced Meaning in Life Scale), perceived social supports (Multidimensional Scale of Perceived Social Support), alcohol use (Alcohol Use Disorders Identification Test), drug use (Drug Abuse Screening Test Short Form 10), problem-solving skills (Social Problem-Solving Inventory–Revised Short Form), and self-reported healthcare costs, as well as health service use measured using Ontario administrative health data. A process evaluation will also be conducted following study completion.

**Discussion:**

The cohort study will test whether better adherence to the intervention results in better outcomes. The value of the cohort study design is that we can examine in more detail certain subgroups or other variables that are not available in the larger cluster randomised trial. This trial will aim to improve standards by informing best practice in management of men who self-harm and present to hospitals in Ontario.

**Trial registration:**

ClinicalTrials.gov, NCT03473535. Registered on March 22, 2018.

## Administrative information

*Note*: The numbers in curly brackets in this protocol refer to Standard Protocol Items: Recommendations for Interventional Trials (SPIRIT) checklist item numbers. The order of the items has been modified to group similar items (*see* http://www.equator-network.org/reporting-guidelines/spirit-2013-statement-defining-standard-protocol-items-for-clinical-trials/).

**Table:** 

Title {1}	The BEACON Study: Protocol for a cohort study as part of an evaluation of the effectiveness of smartphone-assisted problem-solving therapy in men who present with intentional self-harm to Emergency Departments in Ontario
Trial registration {2a and 2b}.	ClinicalTrials.gov, NCT03473535. Registered on March 22, 2018; https://clinicaltrials.gov/ct2/show/NCT03473535
Protocol version {3}	Version 4, March 13, 2019
Funding {4}	The trial is sponsored by the Ottawa Hospital Research Institute (OHRI) and funded by the Ontario Strategy for Patient-Oriented Research (SPOR) Support Unit (OSSU), funded through the Canadian Institutes of Health Research (CIHR) and the Government of Ontario.
Author details {5a}	^1^ Ottawa Hospital Research Institute ^2^ University of Ottawa ^3^ Lawson Health Research Institute ^4^ Western University ^5^ Queen’s University ^6^ Kingston Health Sciences Centre ^7^ Canadian Association for Suicide Prevention ^8^ Sioux Lookout Meno Ya Win Health Centre ^9^ St. Michael’s Hospital ^10^ University of Toronto ^11^ William Osler Health System ^12^ McMaster University ^13^ Carleton University ^14^ Lakehead University ^15^ Institute for Clinical Evaluative Sciences ^16^ Sunnybrook Research Institute ^17^ Wayne State University
Name and contact information for the trial sponsor {5b}	Trial Sponsor: Ottawa Hospital Research Institute (OHRI) Sponsor’s reference: 20150765 Contact name: Dr. Duncan Stewart Address: 501 Smyth Road, Ottawa, ON K1H 8 L6, Canada Telephone: (613) 798–5555, ext.79017 Email: djstewart@ohri.ca
Role of sponsor {5c}	The funders had no role in the review or approval of the manuscript for publication.

## Introduction

### Background and rationale {6a}

In a linked separate paper, we have described the study protocol for a cluster randomised controlled trial (cRCT) to test the effectiveness of a new service that delivers blended problem-solving therapy (PST) compared with usual care (UC) in men aged 18 years or older who present to emergency departments (EDs) in Ontario with intentional self-harm. In the cRCT, 25 EDs were randomised to either UC or the implementation of a smartphone-assisted PST service for men who present to the ED for an episode of self-harm. All outcomes in the cRCT will be obtained from provincial administrative health databases, with the primary outcome being a composite measure of the proportion of men re-presenting to the ED with self-harm and who die by suicide within 1 year of their index presentation. This protocol outlines a cohort study designed to assess the impact of a blended therapy intervention on men enrolled at the intervention sites of the cRCT.

Self-harm has a strong association with suicide: 1.6% of people presenting to EDs with self-harm will die by suicide within 1 year (95% confidence interval, 1.2–2.1%), with the incidence rate in men being almost double the rate in women (2.7% vs. 1.2%) [1]. After 5 years, approximately 3.9% of individuals who have presented to the ED for the treatment of self-harm die by suicide [1]. This risk is more than 50 times greater than the general population rate and is associated with a 40-year reduction in average life expectancy [1]. A recent retrospective study of individuals who died by suicide in southwestern Ontario identified a history of self-harm in more than one-third of decedents [2]. Because presentation to the ED with self-harm is a major identifiable risk factor for suicide, with at least one-fourth of deaths by suicide being preceded by a hospital visit due to non-fatal self-harm in the previous year [3, 4], it is likely that any reduction in the repetition of self-harm will be mirrored by a decline in subsequent deaths by suicide. The Canadian Association for Suicide Prevention Blueprint for a Canadian National Suicide Prevention Strategy has also identified people who have attended hospital because of non-fatal self-harm as a high-risk group to target in order to prevent suicide [5].

Mortality of non-suicidal causes is also high for those who self-harm, with significantly more than the expected numbers of deaths of natural causes and due to accidents [6]. A population-based cohort study investigating administrative datasets in the province of Ontario found all-cause mortality following a first episode of self-poisoning was 1107 per 100,000 person-years, a rate 5.5 times that of the control population. Nearly half of all deaths in this study were attributed to suicide (23.4%), accidents (16.4%), or undetermined intent (5.4%) [7]. Approximately 10% of those who present in an ED following self-harm will engage in repeat self-harm in the following month, and up to 27% will do so after 6 months [8]. Recurrent self-harm is associated with significant distress and many unresolved interpersonal problems [9]. Individuals who self-harm are also frequent users of health and social services [10, 11]. For instance, Morrison & Laing [12] reviewed death records of Alberta patients aged 25 to 64 who died by suicide to develop healthcare use profiles of those who died by suicide compared with those who died of other causes. They found that those who died by suicide averaged more than twice the number of health service visits per person when compared with those who died of other causes [12]. Similarly, those who died by suicide were more likely to have had an ED visit, inpatient hospitalisation separation, or community mental health service than those who did not die by suicide [12].

Numerous studies have also shown that mainstream media reports on suicide influence some people in the general population to engage in self-injury and die by suicide through a copycat phenomenon known as the “Werther effect” [3–16]. This has not specifically been described in males who present to the ED after an episode of self-harm, and the potential impact of the Werther effect was observed with roughly a 10% increase in suicide deaths across the entire United States in the months following the death of the actor and comedian Robin Williams [17]. We will assess exposure to media reports on suicide and access to the internet to inform methods. This could inform future prevention efforts at a population level.

The intervention we will study aims to address this gap and build on previous work, extending the range and intensity of PST by supplementing it with a sophisticated smartphone application and electronic case management that has already demonstrated effectiveness in men with substance abuse disorders [18]. The addition of electronic support to psychotherapy has been called “blended care,” referring to the combination of online and face-to-face therapy in one treatment [19]. Only a small number of studies have investigated blended care, and none has examined blended care in patients who are at high risk for suicide. Large trials in routine clinical settings are needed to assess the effectiveness of blended care interventions in those with mental disorders. In recognition of this, the European Commission has funded a large study, the European Comparative Effectiveness Research on Internet-Based Depression Treatment project (E-COMPARED), in which the effectiveness of blended care for treating depression will be assessed in a randomised controlled trial (RCT) in eight European countries [20]. However, the E-COMPARED study specifically excludes suicidal participants and those with co-morbid mental disorders, such as bipolar disorder or substance abuse.

The present cohort study will assess the impact of treatment with smartphone-assisted PST on suicidal thoughts and behaviour in men over the age of 18 years who present to an ED with intentional self-harm.

The primary objective of this study is to examine the relationship between the number of blended PST sessions attended by men who present to the ED with self-harm and suicidal ideas in the year following their enrolment in the study. Secondary outcomes include health service use (including re-presentation to hospital with self-harm), change in measures of depressive symptoms, anxiety symptoms, symptoms of post-traumatic stress disorder, health-related quality of life, meaning in life, social supports, and substance misuse.

We will also examine the effect on outcomes of the following confounding variables: conformity to “masculine” gender norms, self-reported exposure to reports of suicide in the media, and whether participants searched the internet for methods of suicide for their index presentation.

We will also investigate a theoretical process of change by measuring problem-solving skills assessed by the means–ends problem-solving procedure and the Social Problem-Solving Inventory–Revised Short Form (SPSI-R:S).

### Objectives {7}

This study’s research objectives, primary and secondary research questions, and associated hypotheses are outlined in Tables 1 and 2.

**Table 1:** Primary objective, research question, and hypothesis

Objective	Research question	Testable hypothesis
To evaluate the relationship between the amount of smartphone-assisted PST and suicidal ideas in men over a 1-year period.	In men who present to the ED with self-harm, is the amount of smartphone-assisted PST linked to a reduction in suicidal ideas in the year following their enrolment in the study?	Reduction in severity of symptoms: Suicidal ideation at 1 year will decrease compared with the time of enrolment, proportionally with the number of PST sessions completed by participants. A participant who attends all PST session should experience a greater decrease in suicidality than a participant who does not attend all PST sessions.

*ED* Emergency department, *PST* Problem-solving therapy

**Table 2:** Secondary objectives, research questions, and hypotheses

Objective	Research question	Testable hypothesis
To evaluate the relationship between the amount of smartphone-assisted PST and health-related outcomes in men who present to ED with intentional self-harm.	After 1 year, what is the effect of the amount of smartphone-assisted PST on the following?	That there will be a significant correlation between the number of completed sessions of smartphone-assisted PST and the following:
	• Depression symptoms	• Reductions in the severity of depression symptoms
	• Anxiety symptoms	• Reductions in the severity of anxiety symptoms
	• PTSD symptoms	• Reductions in the severity of PTSD symptoms
	• Healthcare costs	• Reductions in healthcare costs
	• Experienced meaning in life	• Improvements in experienced meaning in life
	• Perceived social supports	• Improvements in perceived social supports
	• Health-related quality of life	• Improvements in health-related quality of life
To evaluate the impact of conformity to masculine gender norms and explore to suicide in the media on men who self-harm	What is the effect of the following?	
	• Conformity to masculine gender norms	Conformity to masculine gender norms will moderate the effect of the study intervention.
	• Exposure to suicide in the media	Exposure to suicide in the media will moderate the effect of the study intervention.
	• Use of the internet to research means of self-harm	Use of the internet to research means of self-harm will moderate the effect of the study intervention.
	• Use of the internet to access self-harm resources	Use of the internet to access self-harm resources will moderate the effect of the study intervention.
To evaluate mechanisms of change among men who self-harm	What are the mechanisms of change among men enrolled in the study?	There will be a significant correlation between the number of smartphone-assisted PST sessions and improvements in social problem-solving skills.

*ED* Emergency department, *PST* Problem-solving therapy, *PTSD* Post-traumatic stress disorder

### Trial design {8}

This is a cohort study embedded in a cRCT in which the unit of randomisation is hospital EDs in Ontario. This protocol has been developed according to the Standard Protocol Items: Recommendations for Interventional Trials (SPIRIT) statement.

## Methods: participants, interventions, and outcomes

### Study setting {9}

This study protocol describes a cohort study embedded in a multicentre cRCT that will be conducted at ten sites in Ontario, Canada. Once site allocation is completed, the principal investigator (PI) (SH) will contact members of the department of psychiatry and department of emergency medicine at each intervention site to be site co-PIs to assume responsibility for all study-related activities at their respective site. The PI will then travel to each study site in order to present information about the study in a rounds-style presentation and meet with key stakeholders who will be responsible for the implementation of the study.

### Eligibility criteria {10}

Eligible participants will be men aged 18 years or older who present to EDs in the intervention arm of the cRCT with intentional self-harm. They will be eligible regardless of whether they are hospitalised or discharged during the index ED visit (Table 3).

**Table 3:** Participant eligibility criteria

Inclusion criteria	
1.	Identifies as male.
2.	18 years of age or older
3.	Has presented at a participating ED with self-harm within the preceding 4 weeks. Self-harm is defined as self-reported intentional self-poisoning or self-injury, regardless of whether there is evidence that the act was intended to result in death. Where there is ambiguity about intent, inclusion will be guided by discussion with the PI to allow consistency between sites.
4.	Able to read and understand English or French
5.	Willing to attend six PST sessions for a period of up to 6 weeks
6.	Willing to use a smartphone application to facilitate the treatment of self-harm
7.	Willing to return to hospital for follow-up appointments
8.	Willing and able to provide informed consent

*ED* Emergency department, *PI* Principal investigator, *PST* Problem-solving therapy

There are no exclusion criteria beyond the opposite of the inclusion criteria. Participants are not required to have a smartphone with a data plan in order to participate. Participants who do not have a smartphone with a data plan will be provided with one for a period of 1 year from the date of their study enrolment.

### Who will obtain informed consent? {26a}

All informed consent discussion will be completed by a site-delegated study staff member, such as a research coordinator or research assistant.

### Additional consent provisions for collection and use of participant data and biological specimens {26b}

On the consent form, participants will be asked if they agree to use of their data should they choose to withdraw from the trial. Participants will also be asked for permission for the research team to share relevant data with people from the universities taking part in the research or from regulatory authorities, where relevant. This trial does not involve collecting biological specimens for storage.

## Interventions

### Explanation for the choice of comparators {6b}

Neither a systematic review nor a large multicentre study found a clear relationship between the nature and intensity of standard hospital care and subsequent fatal or non-fatal repetition of self-harm [1]. Given this, rather than randomising participants to a control group, we opted to use a within-subject design in which participants act as their own control subjects. To assess whether completion of PST results in decreased suicidal thoughts and behaviours, we will evaluate changes in suicidality from baseline throughout the course of the study using repeated-measures analysis of variance (ANOVA).

### Intervention description {11a}

#### Intervention: problem-solving therapy

PST is a cognitive behavioural therapy that aims to teach a cognitive skill to improve people’s ability to cope effectively with both minor (e.g., chronic daily problems) and major (e.g., traumatic events) stressors. The use of PST following self-harm is supported by the literature, which suggests that those who self-harm often struggle with how to problem-solve [21–23]. The major treatment goals of PST include (1) the adoption of a positive attitude toward problems in living (e.g., optimism, positive self-efficacy, acceptance that problems are a common life occurrence) and (2) the effective implementation of specific problem-solving behaviours (e.g., recognising problems when they occur and the ability to stop and think about potential solutions) [24].

#### BEACON prescription management system

##### Smartphone application

The original version of this smartphone application was tested in an RCT in male veterans in the United States [18] and was found to be effective in reducing harmful substance use. It has been redesigned for the purpose of this study to facilitate the treatment of self-harm in men who present to the ED. The sections of the smartphone application are outlined in Table 4.

**Table 4:** Process evaluation outcomes

Data collection method	Data collected
Program documentation and observation (to assess fidelity, dose, reach, and context)	Number of PST sessions attended
	Smartphone application use, including total number of mood log entries, surveys completed, journal entries, goals completed, views/downloads of resource material, and BEACON button presses
	Whether each site implemented other hospital-based suicide reduction measures during the study intervention period
Structured qualitative interviews (to assess barriers, facilitators, and suggestions for improvement)	Interview a purposive sample of participants regarding what helped and what did not help and the effect of the intervention on help-seeking behaviours

*PST* Problem-solving therapy

##### Clinician dashboard

Clinicians will have access to a clinician dashboard where they can see their patient lists of people using the smartphone app. Through this dashboard, they can push resources to patients, see when they have activated their BEACON button, initiate survey measures, and manage appointments and program reminders for goals agreed in face-to-face therapy.

#### Timing of sessions

Men who present with intentional self-harm to an ED will be offered, in addition to UC, six sessions of PST with a “booster” session at 6 months supplemented by the BEACON prescription management system. The initial six sessions will be delivered in an 8-week window after the index presentation to allow some flexibility in the timing of the therapy.

### Criteria for discontinuing or modifying allocated interventions {11b}

The cohort study will be stopped if there are more than 12 deaths per year.

### Strategies to improve adherence to interventions {11c}

The use of a blended therapy approach with the addition of the BEACON prescription management system to face-to-face PST may increase adherence to the face-to-face sessions. The patient-facing smartphone application was previously identified as increasing feelings of connectedness in participants between face-to-face sessions [25]. To improve adherence to the use of the BEACON prescription smartphone application, the application has included daily activities to encourage daily use, as well as the option to set reminders by either the clinician or the participant, which will push notifications to the participant’s phone, serving as an additional prompt to continue using the application.

### Relevant concomitant care permitted or prohibited during the trial {11d}

Not applicable; participants will continue to receive UC while enrolled in this study.

### Provisions for post-trial care {30}

In the event of a study-related injury or illness, participants will be provided with appropriate medical treatment and care. Financial compensation for lost wages, disability, or discomfort due to an injury or illness is not generally available.

### Outcomes {12}

#### Primary outcome

The primary outcome measure will be changes in the severity of suicidal ideation, as measured by the Beck Scale for Suicide Ideation (BSS). This is a 24-item self-report questionnaire used for detecting and measuring the current intensity of participants’ attitudes, behaviours, and plans to die by suicide during the past week. The BSS has strong internal consistency (α = 0.89), has been found to be significantly correlated with the suicide ideation item on the Beck Depression Inventory [26], and is a strong predictor of admission to hospital for managing suicide risk [26].

#### Secondary outcomes

##### Depressive symptoms

Changes in depression severity will be assessed using the Patient Health Questionnaire (PHQ-9), a nine-item questionnaire that assesses the severity of self-rated depression symptoms experienced within the last 2 weeks. Participants are asked to rate each symptom of depression on a frequency scale from 0 (not at all) to 3 (nearly every day), with total scores ranging from 0 (minimal depression) to 27 (severe depression). The PHQ-9 has an internal consistency of 0.89 and strong test–retest reliability [27].

##### Anxiety symptoms

Changes in anxiety symptom severity will be assessed using the GAD-7 (Generalized Anxiety Disorder 7-item scale), a 7-item questionnaire that assesses the severity of generalised anxiety disorder symptoms experienced within the last 2 weeks. The initial validation study, conducted by Spitzer et al. [28], demonstrated high internal consistency (α = 0.92) and test–retest reliability (intraclass correlation coefficient, 0.83) [28].

##### Post-traumatic stress disorder symptoms

Changes in post-traumatic stress disorder (PTSD) symptoms will be evaluated using the Primary Care PTSD Screen, which consists of four items that evaluate the presence of PTSD-related symptoms. The screening tool was initially developed and validated in a population of male and female veterans in a primary care setting [29]. Prins et al. [29] recommended using a cut-off score of 3 (out of a possible 4 points) to detect possible PTSD with a sensitivity of 0.78 and specificity of 0.87.

##### Health-related quality of life

Health-related quality of life will be assessed using the EuroQol 5-dimension 5-level questionnaire (EQ-5D-5L). This is a five-item questionnaire that assesses health-related quality of life, including mobility, self-care, ability to participate in one’s usual activities, pain or discomfort, and anxiety or depression. The measure also includes a visual analogue scale that asks participants to evaluate their overall health on a scale from 0 to 100.

##### Meaning in life

Previous research has demonstrated perceived “meaning in life” to be negatively associated with depression and suicidal ideation [30]. In the present study, meaning in life will be evaluated using the Experienced Meaning in Life Scale [30], which consists of four 10-item subscales: creative, experiential, attitudinal, and ultimate meaning in life. This measure was developed to be consistent with Frankl’s treatment of the construct, was validated in a community-based sample of older adults [30], and has high internal consistency (α = 0.95). For the purposes of this study, we will use only the creative and attitudinal subscales in order to reduce the burden on participants and investigators.

##### Perceived social support

Perceived social support will be assessed using the Multidimensional Scale of Perceived Social Support (MSPSS) [31]. The MSPSS is a 12-item questionnaire that addresses the following sources of perceived social support: family, friends, or significant others. Each subscale consists of four items that are rated on a 7-point Likert scale from “very strongly disagree” to “very strongly agree”. The MSPSS performs well psychometrically with high internal consistency and test–retest reliability.

##### Alcohol misuse

Alcohol misuse will be evaluated using the Alcohol Use Disorders Identification Test (AUDIT). Participants will initially complete the first three items of this questionnaire assessing potential alcohol misuse; those who score above 4 on these items will be asked to complete the remaining seven items. Among those known to misuse alcohol, the AUDIT successfully detected an alcohol use disorder 99% of the time [32]. Similarly, among those who did not misuse alcohol, only 0.5% were categorised as potentially having an alcohol use disorder [32].

##### Drug misuse

Drug misuse will be measured using the Drug Abuse Screening Test (DAST-10), a ten-item questionnaire that assesses drug abuse within the last 12 months. Participants are asked to answer ten questions about their substance use with a binary response of yes or no, with each response indicating a possible drug use problem being awarded 1 point. The total possible scores on this instrument range from 0 to 10, with higher scores indicating a greater likelihood of a substance use problem. The DAST-10 has been evaluated among psychiatric patients and has been found to have high internal consistency (α = 0.94) [33] and a test–retest reliability score of 0.71 [34]. Scores on the DAST-10 have been found to be significantly correlated with the frequency of drug use (*r*-value ranging from 0.19 to 0.55).

##### Health service use

Health service use 12 months after the index presentation will be captured using routinely collected administrative health data housed at the Institute for Clinical Evaluative Sciences (IC/ES) linked to participants’ Ontario Health Insurance Plan (OHIP) numbers. This will include hospitalisations for self-harm, presentation to hospital for self-harm, presentations to hospital for any reason other than self-harm, admission to hospital for any reason, outpatient appointment for any reason, and primary care visits. We will collect this information for all participants, regardless of whether they complete their treatment.

We will also administer a health care cost Questionnaire that has been created by the research team to capture health service use data not available in IC/ES. Specifically, it includes elements from the Client Service Receipt Inventory and the Work Productivity and Activity Impairment Questionnaire: Specific Health Problem as well as other health cost indicators.

#### Confounding variables

##### Adherence to masculine gender roles

The Conformity to Masculine Norms Inventory (CMNI) [35] is a 94-item questionnaire that assesses the conformity to the following masculine gender norms: winning, emotional control, risk taking, violence, power over women, dominance, playboy, self-reliance, primacy of work, disdain for homosexuals, and pursuit of status. The CMNI has strong measures of internal consistency across all 11 subscales (α ranging from 0.72 to 0.91) [35]. It is also strongly correlated with other measures of masculinity, including the Brannon Masculinity Scale [36], the Gender Role Conflict Scale [37], and the Masculine Gender Role Stress Scale [38]. For the purposes of this study, only the emotional control (CMNI-EC) and self-reliance (CMNI-SR) subscales will be used, because they are the most applicable to the study population. This will also reduce participant and investigator burden.

##### Influence of the media and internet use

In order to assess the potential impact of the media and the internet on self-harm presentations, each participant will be asked if, in the month prior to his index self-harm episode, he was (1) aware of any high-profile suicides in the news, (2) whether he used the internet to research self-harm methods, and (3) whether he used the internet to get access to help for his emotional distress.

#### Assessing potential processes of change

##### Problem-solving skills

In order to assess the impact of the smartphone-assisted PST intervention, we will be assessing participants’ social problem-solving skills throughout the study intervention period using the SPSI-R:S [39]. This is a 25-item questionnaire that assesses an individual’s strengths and weaknesses in problem-solving abilities and is a reliable and valid instrument for assessing problem-solving abilities [39].

#### Process evaluation

### Participant timeline {13}

### Sample size {14}

The number of sites for the BEACON (Smartphone-Assisted Problem-Solving Therapy in Men Presenting to the ED with Self-Harm) study was determined by the cRCT. Using IC/ES data, we determined that identifying a cohort of men who present with intentional self-harm over the course of 1 year and then following up with them for 12 months at 10 intervention sites and 15 control sites in Ontario would give us 80% power to detect a relative reduction in repetition rate of 41% (i.e., from a control arm rate of 13% to an intervention arm rate of 7.7%). For this substudy embedded within the intervention arm of the trial, we anticipate that we will be able to recruit 20 to 50 men at each of the intervention sites with an average of 35 men per site, for an anticipated total of 350 men. To test our primary hypothesis that the severity of suicidal thinking at 1 year will decrease with the number of PST sessions completed by participants, we will use both linear and dichotomous modelling approaches. For linear correlations, we will have 80% power to detect a correlation of *r* = 0.15 between number of PST sessions and severity of suicidal thinking measured by the BSS. For the dichotomous approach, we will divide participants into those who have received fewer than three sessions (zero to two sessions) and those who have received three or more sessions. The rationale for this is that three sessions of PST is the minimum number to complete one “cycle” of problem solving. Previous studies have found a mean score of 18 on the BSS for individuals who present with self-harm [40]. To achieve 80% power to detect an effect size of 0.3, or an absolute decrease of 2.4 points from a mean of 18 points on the BSS, we will require 350 participants. Assuming the ratio of those with three or fewer sessions to those with more than three sessions is between 0.75 and 1.25, we anticipate 175 in each group (e.g., 0–2 sessions/3+ sessions). This assumes a common standard deviation on the BSS of 7.9 points based on a comparison of suicidal and non-suicidal samples of adults receiving psychiatric care [40].

### Recruitment {15}

Men who present with intentional self-harm to an ED that has been randomised to receive the study intervention will be approached by ED or study staff with information about the study. A delegated study staff member at each site will also routinely review the electronic medical records at their respective site to ensure that no potentially eligible patients have been missed. These patients will be provided with information about the study by telephone. Patients interested in participating in the study will be scheduled for a baseline intake appointment with a delegated study staff member. We will manage the transition from the ED to outpatient care by providing staff training, written information for patients, and an electronic referral service at each site.

At their baseline intake appointment with a research assistant, eligible participants will download the BEACON prescription smartphone application, and they will be introduced to the on-boarding process and set-up of their profile. Once this is complete, participants will be asked to complete the baseline intake assessments and will be referred for their first PST session. In order to limit participant burden, they will have the choice either to complete their baseline visit and first PST session in one study appointment or to split these visits into two study appointments.

## Assignment of interventions: allocation

### Sequence generation {16a}

This is a cohort study embedded in a cRCT, so no randomisation will take place.

### Concealment mechanism {16b}

Not applicable.

### Implementation {16c}

Not applicable.

## Assignment of interventions: blinding

### Who will be blinded? {17a}

There is no blinding in this study, because all patients who consent to participate in this study will receive the study intervention.

### Procedure for unblinding if needed {17b}

Not applicable.

## Data collection and management

### Plans for assessment and collection of outcomes {18a}

All outcome measures will be administered via paper-based questionnaire at the baseline visit. Questionnaires will then be administered via the BEACON suicide prevention smartphone application as per Table 5.

**Table 5:**
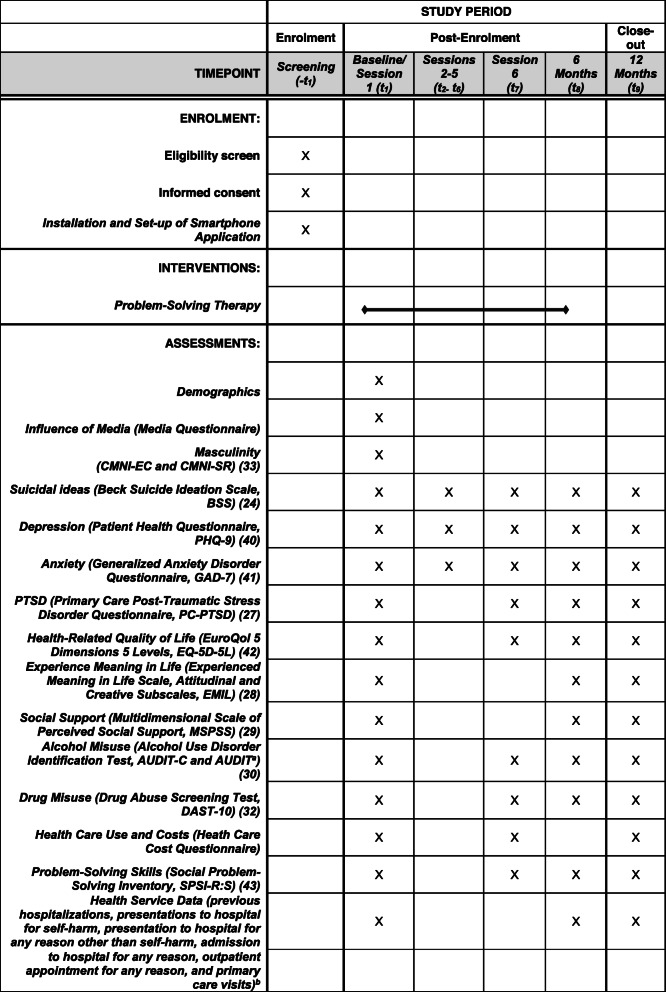
Schedule of enrolment, interventions, and assessments

### Plans to promote participant retention and complete follow-up {18b}

A major difficulty in all clinical trials is ensuring that patients attend their baseline appointment. As such, a key component to our patient recruitment strategy is to ensure minimal loss to follow-up between the point of referral and study enrolment. One way that we are addressing this is through the use of an electronic referral system in which referring clinicians can schedule a baseline intake appointment with interested patients while still in the ED. The goal is to schedule these appointments within 1–2 business days of the presentation.

Once enrolled in the study, standard procedures for loss to follow-up will be followed for patients who do not complete their follow-up appointments. This is an escalated response that may include any combination of the following: continued attempts to contact the participant by postal mail, telephone, and/or email; contacting a participant’s emergency contact; and contacting a participant’s family physician.

The BEACON suicide prevention smartphone application will also be designed to increase participant engagement with the study. For instance, participants will have instant and time-delayed communication with their research therapist through the messaging feature of the mobile application. Research therapists will also have access to a clinician dashboard that will allow them to identify patterns of use which might indicate that a participant may be at risk of being lost to follow-up (i.e., through decreased use of the smartphone application). This will provide research therapists with an opportunity to reach out to participants and attempt to re-engage them in the intervention.

### Data management {19}

All data, with the exception of the BSS and SPSI-R:S, will be completed directly by participants using a centralised electronic data capture (EDC) system, developed and maintained by the Ottawa Methods Centre. Participants will create a unique login to the system and will complete the questionnaires remotely or on-site at the time of their visit. The BSS and SPSI-R:S will still be completed in paper format due to unavailability of an electronic license. On a monthly basis, each site will upload their de-identified study data via a secure network OHRI SharePoint folder (password protected and encrypted) to the research coordinator via email as per N2 Standard Operating Procedure 106 File Transfer and its associated OHRI addendum. Data exported from the EDC will be combined with the uploaded site data and combined into a single study master database in IBM SPSS Statistics software (IBM Corp., Armonk, NY, USA). This database will be stored on the OHRI hospital server at the coordinating site, and only the trial management committee (TMC) will have access to it.

All hard copies of original study documentation will be stored in the participant research charts, which will be categorised in numerical order, according to sequential numbering (i.e., 001 to 350). Data collection via the EDC will be by direct entry, and no paper copy will exist. Once all data monitoring, validation, and cleaning activities are complete, an export of the final EDC database as well as any paper records will be archived at a secure storage facility for a period of 10 years, as required by International Conference on Harmonisation of technical requirements for registration of pharmaceuticals for human use good clinical practice (ICH GCP).

### Confidentiality {27}

All study-related documentation will be double-locked in areas with limited access at the appropriate study site. All participants will be assigned a unique participant identification number, which will appear on all documentation included in a participant’s research chart, including study forms, questionnaires, participant progress notes, and correspondence, in order to maintain participant confidentiality. All correspondence with participants will be de-identified in order to remove names and other potentially identifying information prior to being included in the study chart. All documentation, including eligibility screening forms, signed informed consent forms, and messaging logs, will be stored in double-locked filing cabinets in areas with limited access and stored separately for participant research charts to avoid linking a participant’s name and unique identification number.

Each study site will have a separate master tracking log that will link participants’ names and identification numbers. The combined master tracking log for all study sites will be stored at the coordinating study site. These will be password-protected, and only delegated research staff at that site will have access to it. Participant information will be stored on the secured hospital servers at each study site. The password-protected documents containing participant information will be transferred to the coordinating study site by email, as per N2 and OHRI guidelines. All data are encrypted at rest as well as in transit. Participants’ study information will not be released unless a delegated study staff member obtains written permission from the participant or when required by law. Participants will not be identified in study presentations or publications. All participant records will be kept for a period of 10 years, as indicated in the ICH GCP guidelines.

### Plans for collection, laboratory evaluation, and storage of biological specimens for genetic or molecular analysis in this trial/future use {33}

If this study protocol is amended to include any ancillary studies, upon approval of the REB, all participants involved in these ancillary studies will be asked to sign a consent update form. If a separate informed consent form is required, a copy of the consent form will be stored with the BEACON study consent documentation. Copies of all REB approvals for the ancillary studies will be stored at the coordinating study site. A data file tracking all signed ancillary consent forms must be maintained by the ancillary study and provided to the clinical research coordinator of the BEACON study.

## Statistical methods

### Statistical methods for primary and secondary outcomes {20a}

Categorical participant characteristics, such as gender identity, marital status, and education level, will be reported using descriptive statistics with frequencies and percentages. Continuous characteristics, such as age, will be reported using mean ± SD for continuous variables that are normally distributed and as median and 25th and 75th percentiles for non-normally distributed variables. Non-normally distributed variables will also be dichotomised and analysed as categorical data, as described above. Changes in participants’ scores from their baseline visit to follow-up at 1 year will be by repeated measures ANOVA with generalised linear mixed modelling to account for missing variables. Multivariate linear regression analyses will be performed to determine which participant characteristics moderate primary and secondary treatment outcomes.

### Interim analyses {21b}

An interim analysis will be performed after 9 months by an external party who is blinded to the site allocation. The results of this analysis will be reviewed with the data and safety monitoring committee (DSMC), which will make the appropriate recommendations regarding continuation, modification, or termination of the study.

### Methods for additional analyses (e.g., subgroup analyses) {20b}

Additional subgroup analyses will be carried out to determine the impact of smartphone-assisted PST for the following subgroups: first-time presentations of self-harm compared with repeaters, Francophone versus Anglophone, men with substance abuse disorders versus no substance abuse disorders, and rural versus urban residence.

#### Process evaluation

We will also conduct a process evaluation to explore the implementation, receipt, and context of the intervention with a view to helping understand the results in accordance with the Medical Research Council’s guidelines on assessing complex interventions [41]. This will describe the processes of the intervention group, provide information about the contexts in which the treatments are delivered, and supply information about the experience of being part of the trial. This will also include an exploration of the uptake of the intervention at various sites, including subgroup analyses of the number of face-to-face sessions completed as well as the extent to which participants used the smartphone application.

### Methods in analysis to handle protocol non-adherence and any statistical methods to handle missing data {20c}

The analysis of the primary outcome will be based on self-report data collected through the BEACON suicide prevention smartphone application. As such, the completeness of the data will be impacted by participant withdrawals. In order to minimise the impact of participant drop-out, withdrawal, and loss to follow-up, the research team will follow up with participants regarding the completion of the study questionnaires at their PST appointments. The smartphone application will also be used to prompt and remind participants to complete the study questionnaires. Should participants be lost to follow-up, a delegated study staff member will follow up with them directly to complete the questionnaires either by telephone or by mail. When possible, a delegated study staff member will attempt to ascertain the reasons for drop-out or withdrawal from participants in order to address any issues within the research team’s control in order to prevent future study withdrawals. As such, it is possible that there may be missing data. Characteristics of participants with missing data will be compared with those of participants with complete data to examine the assumption of missing at random. In the case of substantial missingness (e.g., > 5%), missing outcomes will be imputed using multiple imputation prior to analysis.

### Plans to give access to the full protocol, participant-level data, and statistical code {31c}

Anonymised research data will be deposited in an online repository hosted by the Open Science Framework (OSF; https://osf.io/). OSF is a tool that promotes open, centralised workflows by enabling capture of different aspects and products of the research life cycle, including developing a research idea, designing a study, storing and analysing collected data, and writing and publishing reports or papers. It is developed and maintained by the Center for Open Science, a non-profit organisation founded in 2013 that conducts research into scientific practice, builds and supports scientific research communities, and develops research tools and infrastructure to enable managing and archiving research.

## Oversight and monitoring

### Composition of the coordinating centre and trial steering committee {5d}

#### Principal investigator (SH) and co-principal investigator (SH)

- Design and conduct of the BEACON study
- Preparation of protocol and revisions
- Preparation of study documentation
- Organisation of steering committee meetings
- Publication of study reports
- Participation as members of TMC

#### Steering committee

- Includes: PI (SH), co-PI (MH), and site PIs
- Approval of the final protocol
- All co-PIs at each intervention site will be steering committee members.
- Recruitment of patients and liaising with PI and co-PI
- Reviewing progress of study and, if necessary, approval of changes to the protocol and to facilitate the smooth running of the study

#### Trial management committee

- Includes: PI (SH), co-PI (MH), and clinical research associates (NE, SM)
- Study planning
- Organisation of steering committee meetings
- Organisation of DSMC meetings
- Provide annual reporting to REB
- Serious adverse event (SAE) reporting to DSMC and REB
- Responsible for master tracking log
- Budget administration and contractual issues with individual centres
- Advice for lead investigators
- Coordination of study monitoring
- Assistance with REB applications
- Data verification
- Randomisation

### Composition of the data monitoring committee, its role and reporting structure {21a}

The DSMC is comprised of four members from the following fields of expertise: statistics/biostatistics, epidemiology, methodology, psychiatry, and the ethics of clinical trials.

Its responsibilities are as follows:

- Ensures the ongoing safety of study participants
- Reviews the conduct of the study, including protocol violations and deviations
- Reviews data on participant recruitment, accrual, and retention, as well as assessments of data quality, completeness, timeliness, data retention, data storage, data transmission, and data access
- Reviews adverse events (AEs) and SAEs reported between meeting dates
- Protects the confidentiality of the study data and the DSMC discussions
- Makes recommendations to continue, modify, or terminate the study

### Adverse event reporting and harms {22}

During the active treatment period (baseline visit to session 6), the following occurrences will be routinely collected and assessed by delegated study staff members: death by suicide, subsequent self-harm, visits to the ED or other unscheduled hospitalisations, and re-presentations to the ED for self-harm. All AEs will be reviewed and classified by the site co-PI at his/her discretion. Investigators will determine relatedness of an event to the study intervention based on a temporal relationship to the study intervention, as well as whether the event is unexpected or unexplained, given the participant’s clinical course, previous medical conditions/history, and concomitant medications or interventions. It is estimated that approximately 1% of participants enrolled in the study will die by suicide and 1% of participants will die of causes other than suicide in each year of the study [1]. This results in an anticipated rate of approximately six deaths per year. To address this, the research team has developed standard operating procedures to manage participant suicidality and mitigate potential staff burnout.

Throughout the study, participants will be able to contact the appropriate local mental health crisis teams at any time. This emergency information will be provided to participants during their baseline visit. Suicidal thoughts and self-harm will be monitored closely at each participant follow-up appointment using both the BSS and the Columbia-Suicide Severity Rating Scale [42]. If the participant’s suicidality worsens, this will be recorded as an AE or SAE, as appropriate, and monitored. Participants will also have access to a licensed professional between study visits up until the 6-month study follow-up and will be connected with mental health services as needed. Between the 6- and 12-month follow-up visits, participant activity will be monitored via the clinician dashboard and follow-up by a delegated study staff member as needed.

### Frequency and plans for auditing trial conduct {23}

Following site initiation, an internal monitor will be selected. This monitor will not be involved in data collection activities and will be one step removed from the clinical trial. The internal monitor will perform the first monitoring visit at each site shortly after the site has recruited their first participant to ensure that research personnel have implemented the appropriate recruitment processes and procedures, such as eligibility sign-off and consent. This visit will be completed prior to the site recruiting more participants. Any corrective actions implemented regarding inconsistencies identified during the previous monitoring visits will be assessed for completeness. On the basis of research category and participant/institute risk exposure, remote monitoring visits will occur every month after the first monitoring visit. The internal monitor may schedule more visits or on-site visits as needed.

### Plans for communicating important protocol amendments to relevant parties (e.g., trial participants, ethics committees) {25}

Any subsequent modifications to the study protocol, including changes to study objectives, study design, patient population, sample sizes, study procedures, or significant administrative changes will be agreed on by the steering committee and submitted to the REB for review and approval prior to implementation.

### Dissemination plans {31a}

#### Data analysis and release of results

To protect the scientific integrity of this study, data from all clusters will be analysed and reported together. Although subanalyses with specific groups will be conducted, no centre is expected to report data collected from their centre alone. The primary data analysis will be conducted by the Ottawa Methods Centre at OHRI in conjunction with IC/ES. All statisticians will be blind to the allocation of the study sites. All study publications and presentations are expected to adhere to the BEACON study objectives as detailed in this protocol.

#### Review process

A publications committee, a subcommittee of the steering committee, will be established to coordinate all study publications and presentations. All presentation and publication abstracts must be submitted for review by the publications committee. This committee will create a running list of all potential publications, review all abstracts submitted for publication by the investigative team, identify a lead author for each publication, review all publication manuscripts, and submit publications to peer-reviewed journals for publication. It will also ensure that all publication guidelines and regulations are respected, including adherence to the study’s objectives and the Consolidated Standards of Reporting Trials (CONSORT) statement for cRCTs.

Each presentation or publication abstract/manuscript must be submitted to the research coordinator prior to each publications committee meeting. The abstracts will be reviewed at the subsequent publications committee meeting. All members will vote on each abstract and will provide feedback. The research coordinator will include all feedback in the meeting minutes and, after each meeting, will circulate all feedback appropriately. Authors will be expected to review the committee’s feedback and re-submit their final abstract or manuscript for final approval by the publications committee.

#### Primary outcome publications

The publications committee will ensure that no presentation or publication undermines the dissemination of any primary outcome publications. Primary outcome publications refer to any presentation or publication that presents data on the primary outcomes as detailed in this protocol. During the review process, the publications committee will determine if an abstract/manuscript will undermine any primary outcome publications. If it is determined that this is the case, the author will be asked to delay publication until such time as the primary outcome publication is released.

#### Other study papers, abstracts, and presentations

This refers to all presentations and publications that do not report on the primary outcome of this trial, as detailed in this protocol. All presentation and publications abstracts/manuscripts must be reviewed and approved by the publications committee prior to submission.

#### Close-out procedures

The primary outcome publication is expected to be submitted for publication within 2 years of the completion of collection of follow-up data (i.e., after the last study participant has completed the study). However, this may occur at an earlier or later date if the circumstances warrant. Study close-out will occur in two stages:

1. Period of analysis and documentation of primary outcome results
2. Debriefing of participants and dissemination of all other study results

#### Reporting of study results

All study results will be released to study participants, referring clinicians, patients, and the general medical community. Results will be communicated to study participants through the use of a newsletter or presentation, as per the overall preference of the participants. Other forms of dissemination include academic publications, conference presentations, and presentations to the general public.

SPIRIT guidance: Plans for investigators and sponsor to communicate trial results to participants, healthcare professionals, the public, and other relevant groups (e.g., via publication, reporting in results databases, or other data sharing arrangements), including any publication restrictions.

## Discussion

This study of a combined face-to-face and technological intervention will provide information about the acceptability and use of the therapy in a high-risk group of men. It will also inform the practice of how to use technology in face-to-face psychotherapy. Important operational issues are privacy and confidentiality of any information stored on the app. The app contains only data that the user chooses to enter, such as messages to the therapist. Users can also choose to anonymise their presence by choosing any username. Last, the app has undergone security testing by an external company. Given the nature of the trial, which involves mental health care in a high-risk group using technology, regulatory approval has been lengthy, which means that operational platforms such as iOS have changed during the trial set-up. This is to be expected but adds to the cost and complexity of the technological application. However, the combination of a patient-facing app and a clinician-facing dashboard based on problem-solving skills has resulted in a system that is now suitable for use in supporting management of other chronic disorders.

## Trial status

This study received initial approval by the OHSN-REB in January, 2018. It is anticipated that all sites will be initiated within the next three to six months. At the time of manuscript submission, no participants had been enrolled in the study. The estimated completion date for primary data collection is March, 2021.

## Supplementary information

**Additional file 1.** IC/ES databases and descriptions.

## Data Availability

Any data required to support the protocol can be supplied upon request.

## Abbreviations

AE: Adverse event
ANOVA: Analysis of variance
AUDIT: Alcohol Use Disorders Identification Test
BDI: Beck Depression Inventory
BSS: Beck Scale for Suicide Ideation
CIHR: Canadian Institutes of Health Research
CMNI: Conformity to Masculine Norms Inventory
CMNI-EC: Conformity to Masculine Norms Inventory, emotional control subscale
CMNI-SR: Conformity to Masculine Norms Inventory, self-reliance subscale
cRCT: Cluster randomised controlled trial
CTO: Clinical Trials Ontario
DAST-10: Drug Abuse Screening Test Short Form 10
DSMC: Data and safety monitoring committee
E-COMPARED: European Comparative Effectiveness Research on Internet-Based Depression Treatment project
ED: Emergency department
EDC: Electronic data capture
EQ-5D-5 L: EuroQol 5-dimension 5-level questionnaire
GAD-7: Generalized Anxiety Disorder 7-item scale
IC/ES: Institute for Clinical Evaluative Sciences
ICH GCP: International Conference on Harmonisation of technical requirements for registration of pharmaceuticals for human use good clinical practice
MSPSS: Multidimensional Scale of Perceived Social Support
NACRS: National Ambulatory Care Reporting System
OHIP: Ontario Health Insurance Plan
OHRI: Ottawa Hospital Research Institute
OHSN-REB: Ottawa Health Science Network Research Ethics Board
OSF: Open Science Framework
OSSU: Ontario Strategy for Patient-Oriented Research Support Unit
PI: Principal investigator
PTSD: Post-traumatic stress disorder
PHQ-9: Patient Health Questionnaire-9
PST: Problem-solving therapy
RCT: Randomised controlled trial
REB: Research ethics board
SAE: Serious adverse event
SD: Standard deviation
SPIRIT: Standard Protocol Items: Recommendations for Interventional Trials
SPOR: Ontario Strategy for Patient-Oriented Research (SPOR)
SPSI-R:S: Social Problem-Solving Inventory–Revised Short Form
TMC: Trial management committee
UC: Usual care
