# Supplementary material for: The BEACON study: protocol for a cohort study as part of an evaluation of the effectiveness of smartphone-assisted problem-solving therapy in men who present with intentional self-harm to emergency departments in Ontario
Source: Trials. 2020 Nov 13;21:925. doi: 10.1186/s13063-020-04424-w (PMC7663866; doi:10.1186/s13063-020-04424-w)
Supplement: Supplementary file 1 — Additional file 1. IC/ES databases and descriptions. [file 13063_2020_4424_MOESM1_ESM.docx]

**Additional File 1 – IC/ES Databases and Descriptions**

| **Acronym** | **Database Name** | **Database Description** |
| --- | --- | --- |
| NACRS | National Ambulatory Care Reporting System | The National Ambulatory Care Reporting System (NACRS) is a data collection tool used to capture information on patient visits to hospital and community based ambulatory care: day surgery, outpatient clinics and emergency departments. |
| OHIP | Ontario Health Insurance Plan Claims Database | The Ontario Health Insurance Plan (OHIP) claims data contains most claims paid for by the Ontario Health Insurance Plan. The data cover all health care providers who can claim under OHIP (this includes physicians, groups, laboratories, and out-of-province providers). |
